# Effect of the Optical Zone Ablation Diameter on Higher Order Aberrations After Transepithelial Photorefractive Keratectomy: A Cohort Study

**DOI:** 10.7759/cureus.17630

**Published:** 2021-09-01

**Authors:** Mansour M Al-Mohaimeed

**Affiliations:** 1 Department of Ophthalmology, College of Medicine, Qassim University, Qassim, SAU

**Keywords:** aberration, trans photorefractive keratectomy, myopia, refractive surgery, optical zone

## Abstract

Objectives: To study the effect of the optical zone diameter of ablation on higher order aberrations after transepithelial photorefractive keratectomy for myopia and myopic astigmatism.

Methods: In this historical cohort study in 2019, patients were grouped into 7-mm (Gr-1) and 6.5-mm optical zones of ablation (Gr-2). Topographic and higher order aberrations at analysis diameters of 2, 4, and 6 mm were measured before and six months after transepithelial photorefractive keratectomy. The changes in the five types of higher order aberrations in the 6.5 mm and 7 mm groups were compared. The pupillary diameter was correlated with the change in the higher order aberrations.

Results: We had 24 eyes of 12 patients in Gr-1 and 80 eyes of 40 patients in Gr-2. The trefoil type of higher order aberrations at 6 mm was significantly more prevalent in Gr-2 than in Gr-1 before surgery (p = 0.038). The change in spherical aberration six months after surgery compared with before was significantly more at 6 mm in the eyes of Gr-2 patients (p = 0.02). For the eyes managed by the 7-mm optical zone of ablation for transepithelial photorefractive keratectomy, the decline in the different types of higher order aberrations was significant. The pupillary diameter was positively correlated with the change in the third-order coma in Gr-2 (Spearman coefficient, p = 0.005). All the eyes had an uncorrected visual acuity of 0.0 LogMAR in Gr-1 and 95% in Gr-2 after surgery.

Conclusions: The higher order aberrations six months after transepithelial photorefractive keratectomy were similar in eyes managed with 7-mm and 6.5-mm optical zone for ablation. But a lower aberration coefficient in eyes was managed by the 7-mm zone than the 6.5-mm zone of optical ablation at 6-mm analysis diameter.

## Introduction

The human population is facing a myopia epidemic [1]. A number of options such as spectacles, contact lenses, phakic intraocular lens, and refractive surgeries are available to provide clear vision. These have enabled us to address lower order aberration (LOA) corrections and provide spectacle-free vision [2]. Refractive surgeries have evolved considerably in the last decade, and this has brought the issue of higher order aberrations (HOAs) into focus [3,4]. Patients with successful correction of lower order aberrations resulting in ametropic vision still complain of a less-than-desired quality of vision. Wavefront technology of eye examinations has identified HOAs such as coma, trefoil, and spherical aberration [5]. This eye assessment technology has enabled us to identify the location of HOAs on cornea. Moreover, HOA can be resolved through surgical intervention. Unfortunately, laser-assisted refractive surgeries can also cause HOAs. Hence, it is advised to review the potential risk factors of HOAs before refractive surgeries and manage them to avoid causing additional HOAs unintentionally [6].

Several studies have reviewed the impact of different types of refractive surgeries on HOAs and the role of the optical zone diameter for ablation in causing discomfort due to a change in HOAs. These surgeries include femtosecond laser-assisted surgery, laser in situ keratomileusis, laser-assisted sub-epithelial keratectomy (LASEK), photorefractive keratectomy, and Epi-LASIK (laser-assisted in situ keratomileusis) [2,6,7-10].

The effect of the optical zone diameter selected to perform photorefractive keratectomy excimer laser treatment for myopia has been examined, finding that the 7 mm optical zone provides better outcomes when treating some HOAs [11]. Some refractive surgeries have resulted in change for the better, whereas others have led to undesirable HOAs [8,12,13]. Therefore, it is essential to compare postoperative HOAs with preoperative HOAs in refractive surgeries.

Myopic patients in the Middle East undergo refractive surgery since the climatic conditions are often not conducive for contact lenses. A dry humid climate also poses challenges to patients’ decision to undergo refractive surgeries as tear film abnormalities also can cause HOAs [14].

In this study, we present the comparison of 6.5-mm and 7-mm diameter optical zones on the change in different types of HOAs following T-PRK among patients with myopia and myopic astigmatism and its determinants.

## Materials and methods

Our study is a retrospective, consecutive, and non-randomized cohort study. It included eyes operated by single-step T-PRK from September to December 2019 at a private eye clinic in the Kingdom of Saudi Arabia. The local research and ethics committee approved this study. The tenets of the Helsinki declaration for human research were strictly followed. The patient was adequately informed about the study as well as the risks and benefits of the surgery, and signed informed consent was obtained.

We tested the hypothesis that HOAs are significantly less in T-PRK surgeries performed using the 7-mm optical zone than the 6.5-mm optical zone for ablation [11]. To achieve a 95% confidence interval and 90% power in the cohort study with a ratio of 1:3 of cases and comparison groups with 1.49 ± 0.34 and 1.36 ± 0.38 aberration coefficients as reported by Ozulken et al., we needed a sample size of 120 eyes (30; 90). Hence, we used the sample size determination for health studies software of the World Health Organization [11,15].

The inclusion criteria included age above 18 years, primary myopia or compound myopic astigmatism, preoperative manifest refraction spherical equivalent (MRSE) within the range of −1.50 to −7 D, a stable refraction for at least one year before the surgery, contact lens discontinuation for at least three weeks, and an estimated residual stromal corneal bed thickness of >350 μm at the thinnest location. The exclusion criteria included previous ocular surgery, active ocular diseases, corneal dystrophy, retinal disease, dry eye, a history of severe eye trauma, irregular astigmatism or suspected keratoconus on corneal topography, any previous ocular surgery, glaucoma or glaucoma suspect, diabetes mellitus, autoimmune diseases, pregnancy, breastfeeding, and moderate-to-severe dry eye.

Preoperative information included age, sex, and the eye operated. Patients were instructed not to wear contact lenses for at least 14 days to 21 days before the measurements were to be taken. The uncorrected visual acuity (UCVA) and best-corrected visual acuity (BCVA) were noted for distance using Snellen’s visual acuity chart projected at six meters from the patient. The corneal topography was evaluated using a Pentacam camera (OCULUS-Netzteil Art., Pentacam HR, Germany), and tomography was carried out using Sirius (SCHWIND eye-tech-solutions, GmbH, Kleinostheim, Germany). We used an aberrometer to measure the wavefront aberration to detect the third-order coma value, third-order trefoil value, fourth-order spherical aberration value, aberration coefficient, and Q value. The measurement of each eye included keratometry K1, K2, central corneal thickness, and pupillary diameter in normal daytime illumination in a room. Cycloplegic refraction was performed, and then spherical, cylindrical, and spherical equivalent refractive power in a diopter was noted for each eye.

The ablations were performed using an Amaris 500 Hz excimer laser (SCHWIND eye-tech-solutions, GmbH, Kleinostheim, Germany). The machine was kept in aberration-free mode. The treatment goal was to maintain emmetropia.

Before the surgery, moxifloxacin 0.5% (Vigamox, Alcon Co.) drops were applied. An antiseptic chlorhexidine gluconate 0.05% solution (Saudi Medical Solution Company) was used to clean the eyelids before surgery. Eyes were kept open during surgery using a wire lid speculum.

The application of the laser and other surgical steps are described in detail in the literature [16,17]. The administration of the laser occurred in a single continuous session to ablate both the epithelium and the stroma in a single step using an aberration-free and aspheric profile. According to a population-based epithelium thickness profile, the ablation plan utilized 55 µm centrally and 65 µm peripherally. Eye movements throughout the ablation were compensated by static and dynamic cyclotorsion corrections. Immediately after the ablation, a sponge soaked with 0.02% mitomycin-C was placed over the stromal bed for 25-35 s. Then, we irrigated the eye using copious amounts of balanced salt solution (BSS) (Alcon Laboratories, Fort Worth, TX, USA). A soft bandage contact lens (Bausch & Lomb, New York, USA) with a high-diffusion constant of oxygen permeability was used as a bandage contact lens for six days.

All the measurements relating to distance visual acuity, topography, and HOA were repeated six months after surgery using the same equipment by the same investigators. For each HOA, the values noted six months after surgery were compared with those noted before surgery to determine the change in HOA.

The data were collected using a pretested data collection form. They were collected from both records and a printout of the equipment software files. In cases of disputes, the data in the equipment linked to the case record number were considered correct.

The data were transferred into a Statistical Package for the Social Sciences (SPSS v25) (IBM Corp., Armonk, NY, USA) spreadsheet. The qualitative variables were presented as numbers and percentages. The quantitative variables were plotted to study the distribution. If the distribution was normal, we presented the mean and standard deviation for each group. If the distribution was skewed, we presented their median and interquartile range. To compare the findings of the two groups, we used univariate analysis to estimate the differences in the means, 95% confidence intervals, and two-sided P values based on a matched-pair analysis. To compare the outcomes of the two groups that were not normally distributed, we performed a nonparametric analysis using two related sample calculations and presented the two-sided Wilcoxon P test values. A p-value of <0.05 was considered statistically significant.

## Results

**Figure 1:**
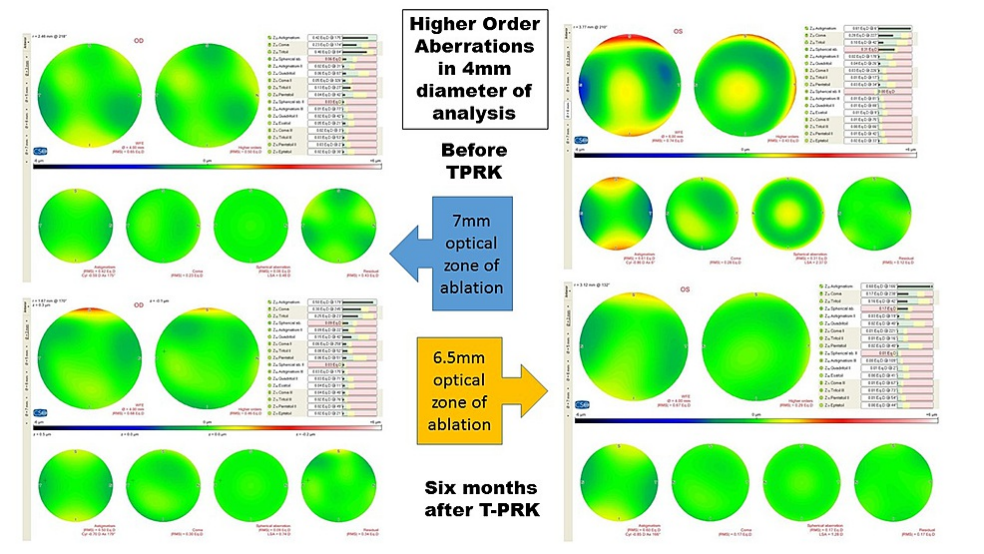
Higher order aberrations in 4-mm diameter of analysis before and after six months of transepithelial photorefractive keratectomy in a patient, each managed by 7-mm and 6.5-mm of optical zone of ablation

Our study comprised 24 eyes of 12 patients (Group 1; Gr-1) managed using the 7-mm optical zone for ablation and 80 eyes of 40 patients (Group 2; Gr-2) managed by the 6.5-mm optical zone of ablation. The mean age of patients in Gr-1 and Gr-2 were 22.9 ± 2.9 years and 25.1 ± 4.1 years, respectively (p = 0.08). There were seven (58.3%) men in Gr-1 and 12 (27.9%) men in Gr-2 (p = 0.009). Nine (37.5%) right eyes in Gr-1 and 43 (53.8%) in Gr-2 were operated (p = 0.17). The HOAs before and after six months T-PRK in 6-mm diameter of analysis of a patient is shown in Figure 1.

The comparison of the topographic parameters and refractive status of the eyes in both groups before T-PRK is given in Table 1.

**Table 1:** Comparison of ocular profile before transepithelial photorefractive keratectomy (T-PRK) in 7-mm and 6.5-mm optical zone of ablation group CCT, Central corneal thickness.

		7.0-mm Optical Zone (24 Eyes/12 Patients)	6.5-mm Optical Zone (80 Eyes/40 Patients)	Validation
Refraction (D) Spherical Cylinder Spherical Equivalents	Median (IQR*)	-3.25 (-4.0; -2.0) -0.5 (-1.0; -0.12) -3.6 (-4.5; -2.3)	-3.0 (-4.25; -2.25) -0.5 (-0.75; -0.5) -3.43 (-4.6; -2.4)	MW P = 0.85; MW P = 0.95; MW P = 0.85
K1	Mean ± standard deviation	42.5 ± 1.3	42.9 ± 1.7	p = 0.209
K2	Mean ± standard deviation	43.6 ± 1.2	44.0 ± 1.2	p = 0.163
CCT	Mean ± standard deviation	538.6 ± 24.7	550.9 ± 36.1	p = 0.063
Pupil diameter	Mean ± standard deviation	6.9 ± 0.33	5.98 ± 0.5	p < 0.001

They were not significantly different. The UCVA of the eyes in Gr-1 before surgery was 0.22 ± 0.21 LogMAR and that of the eyes in Gr-2 was 0.24 ± 0.2 LogMAR (p = 0.69). The BCVA of the eyes in Gr-1 was 0.0 LogMAR and that of the eyes in Gr-2 was 0.0 LogMAR.

The presence of different types of HOAs before T-PRK at 2, 4, and 6 mm diameters from the center of the cornea was compared in Gr-1 and Gr-2 (Table 2). Apart from the third-order trefoil in the 6-mm zone, all the other HOAs were similar in both groups before surgery.

**Table 2:** Comparison of high-order aberration before and after six months of transepithelial photorefractive keratectomy with 7-mm and 6.5-mm optical zone of ablation T-PRK, Transepithelial photorefractive keratectomy.

Higher Order Aberration	Before T-PRK Surgery			Six Months After T-PRK Surgery		
	7.0-mm optical zone (24 eyes of 12 patients)	6.5-mm optical zone (80 eyes of 40 patients)	P value	7.0-mm optical zone (24 eyes of 12 patients)	6.5-mm optical zone (80 eyes of 40 patients)	P value
2-mm diameter of analysis						
Third-order coma	0.08 (0.04; 0.14)	0.09 (0.06; 0.14)	0.334	0.12 (0.06; 0.3)	0.15 (0.07; 0.25)	0.853
Third-order trifoil	0.12 (0.02; 0.2)	0.11 (0.06; 0.2)	0.660	0.17 (0.09; 0.27)	0.15 (0.11; 0.24)	0.754
Fourth-order spherical abrasion	0.03 (0.0; 0.05)	0.06 (-0.04; 0.08)	0.441	0.1 (0.02; 0.18)	0.06 (0.0; 0.12)	0.078
Aberration coefficient	0.07 (0.03; 0.1)	0.06 (0.4; 0.09)	0.997	0.1 (0.07; 0.22)	0.1 (0.07; 0.16)	0.705
Q value	0.09 (0.06; 0.13)	0.14 (0.05; 0.17)	0.389	0.09 (0.04; 0.14)	0.08 (0.05; 0.14)	0.835
4-mm diameter of analysis						
Third-order coma	0.11 (0.08; 0.15)	0.11 (0.05; 0.21)	0.793	0.12 (0.09; 0.16)	0.12 (0.08; 0.18)	0.938
Third-order trifoil	0.11 (0.06; 0.16)	0.13 (0.09; 0.2)	0.076	0.15 (0.10; 0.23)	0.14 (0.09; 0.21)	0.586
Fourth-order spherical abrasion	0.08 (0.06; 0.1)	0.08(0.04; 0.11)	0.718	0.09 (0.04; 0.11)	0.08 (0.05; 0.11)	0.790
Aberration coefficient	0.03 (0.02; 0.04)	0.03 (0.02; 0.05)	0.486	0.05 (0.03; 0.09)	0.04 (0.02; 0.06)	0.222
Q value	0.05 (0.03; 0.08)	0.05 (0.02; 0.09)	0.751	0.06 (0.03; 0.1)	0.04 (0.03; 0.07)	0.195
6-mm diameter of analysis						
Third-order coma	0.16 (0.12; 0.24)	0.17 (0.08; 0.28)	0.969	0.18 (0.09; 0.35)	0.22 (0.12; 0.27)	0.793
Third-order trifoil	0.14 (0.09; 0.19)	0.18 (0.11; 0.25)	0.038	0.17 (0.11;0.29)	0.16 (0.13; 0.24)	0.691
Fourth-order spherical abrasion	0.18 (0.14; 0.23)	0.21 (0.15; 0.24)	0.190	0.25 (0.20; 0.29)	0.28 (0.21; 0.35)	0.128
Aberration coefficient	0.05 (0.03; 0.07)	0.05 (0.02; 0.09)	0.692	0.11 (0.06; 0.15)	0.09 (0.05; 0.12)	0.151
Q value	0.05 (0.03; 0.07)	0.05 (0.03; 0.08)	0.519	0.06 (0.03; 0.09)	0.05 (0.03; 0.07)	0.167

In Gr-1, the mean transition zone of T-PRK was 1.26 ± 0.3 mm, while in Gr-2, it was 1.22 ± 0.38 (p = 0.62). In Gr-1, the mean ablation zone was 7.95 ± 1.86 mm, and in Gr-2, it was 7.6 ± 0.9 mm (p = 0.38). The comparison of HOAs noted six months after T-PRK in Gr-1 and Gr-2 was not statistically different (Table 2).

The change in the value of the HOAs at six months post-T-PRK compared with that noted before surgery was compared in Gr-1 and Gr-2 (Table 3).

**Table 3:** The change in high-order aberration values (six months after T-PRK and before T-PRK) in 7-mm vs. 6.5-mm optical zone groups T-PRK, Transepithelial photorefractive keratectomy.

		7.0-mm Optical Zone (24 Eyes of 12 Patients)		6.5-mm Optical Zone (80 Eyes of 40 Patients)		Validation
		Median	IRQ	Median	IRQ	
Third-order coma	2 mm	0.04	-0.05; 0.14	0.04	-0.01; 0.16	0.60
	4 mm	0.01	-0.05; 0.07	0.00	-0.06; 0.07	0.90
	6 mm	0.03	-0.04; 0.13	0.01	-0.06; 0.08	0.37
Third-order trifoil	2 mm	0.01	-0.06; 0.17	0.04	-0.05; 0.11	0.97
	4 mm	0.02	-0.06; 0.08	0.04	-0.01; 0.1	0.27
	6 mm	0.01	-0.06; 0.05	0.04	-0.02; 0.09	0.13
Fourth-order spherical aberration	2 mm	0.06	-0.02; 0.23	0.03	-0.04; 0.1	0.17
	4 mm	0.00	-0.03; 0.06	0.01	-0.04; 0.02	0.71
	6 mm	0.05	0.00; 0.1	0.10	0.03; 0.17	0.02
Aberration coefficient	2 mm	0.04	0.00; 0.17	0.04	-0.02; 0.09	0.60
	4 mm	0.02	-0.01; 0.03	0.01	-0.01; 0.04	0.58
	6 mm	0.05	-0.01; 0.11	0.03	0.00; 0.08	0.55
Q value	2 mm	-0.01	-0.10; 0.02	0.00	-0.07; 0.04	0.34
	4 mm	0.00	-0.03; 0.03	-0.01	-0.04; 0.03	0.83
	6 mm	0.00	-0.02; 0.04	0.00	-0.03; 0.03	0.67

The change in spherical aberration at 6 mm was significantly more in Gr-2 than in the group of the 7-mm optical ablation zone (Mann-Whitney U test, p = 0.02).

The correlation of the pupillary diameter and change in the HOA values were correlated in Gr-1 and Gr-2 (Table 4).

**Table 4:** Pupillary diameter and change in different HOA values were correlated in 7-mm and 6.5-mm optical zone of transepithelial photo refractive kerectomy for the treatment of myopia

	High-Order Aberration	7-mm Optical Zone of Ablation		6.5-mm Zone of Optical Ablation	
		Spearman Coefficient	P	Spearman Coefficient	P
2 mm	Third-order coma	0.02	0.92	-0.117	0.301
	Third-order trifoil	-0.109	0.612	-0.146	0.196
	Fourth-order spherical abrasion	-0.094	0.663	-0.010	0.928
	Aberration coefficient	-0.299	0.156	0.006	0.703
	Q value	0.128	0.55	-0.077	0.498
4 mm	Third-order coma	0.018	0.933	0.313	0.005
	Third-order trifoil	-0.04	0.85	0.104	0.360
	Fourth-order spherical abrasion	-0.135	0.529	-0.142	0.21
	Abrasion coefficient	-0.086	0.69	0.043	0.703
	Q value	-0.155	0.469	0.016	0.89
6 mm	Third-order coma	-0.014	0.947	0.203	0.07
	Third-order trifoil	-0.198	0.353	0.146	0.195
	Fourth-order spherical abrasion	-0.392	0.06	-0.182	0.107
	Abrasion coefficient	-0.326	0.12	0.034	0.762
	Q value	-0.371	0.074	0.019	0.07

Apart from the third-order coma type of HOA at 4 mm in the 6.5-mm optical ablation zone (Spearman coefficient, p = 0.005), no other HOAs in either group had a significant correlation with the change in HOA due to T-PRK.

All the eyes managed by the 7-mm optical zone for ablation for T-PRK had a UCVA of 0.0 LogMAR at six months. Of the 80 eyes managed by the 6.5-mm optical zone of ablation for T-PRK, 76 (95%) eyes had a UCVA of 0.0 LogMAR and the remaining four (5%) had a UCVA of 0.1 LogMAR.

## Discussion

HOA was noted in all the eyes treated with T-PRK. All the eyes showed a correction of LOAs but still had HOAs to some extent six months after T-PRK. At that time, the HOAs were no different in the eyes treated with the 7-mm and 6.5-mm optical zones of T-PRK. The increase in the fourth-order spherical aberration, however, was significantly more at the 6-mm diameter of analysis if the 6.5-mm optical zone was selected for T-PRK. The pupillary diameter was significantly and positively correlated with the change in the third-order coma in the eyes treated with T-PRK using the 6.5-mm optical zone ablation.

This is perhaps the first study describing the differences between the 6.5-mm and 7-mm optical zones for T-PRK ablation while addressing the HOAs among myopic and myopic astigmatic patients. The selection of a wider optical zone does not make any significant difference when changes in HOAs are desired to compensate for preexisting HOAs. The present study also confirms the need to include wavefront technology to improve vision both for the LOAs and for the HOAs as well as to minimize iatrogenic HOAs in refractive surgery [18].

In the present study, we noted two outcomes: (1) A difference in the HOA values at six months in the 7-mm and 6.5-mm optical zone groups subjected to T-PRK and (2) a change in the HOA values at six months compared with those before T-PRK in these two groups. We did not find any significant difference in the HOA values in the 7-mm and 6.5-mm optical zone groups at six months after T-PRK. By contrast, Ozulken et al. noted significantly fewer spherical aberrations and lower aberration coefficient values in the 7-mm group than those in the 6.5-mm group [11]. The group with the 7-mm optical zone of ablation in the study by Ozulken et al. had lower grades of myopia and a thicker central cornea than those in the 6.5-mm optical zone ablation group. The 7-mm and 6.5-mm optical zone ablation groups in our study had similar grades of myopia and corneal thickness before T-PRK. Thus, preoperative differences in the refractive and topographic profiles of participants in the two studies could explain the differences in HOAs at six months. In a study using a 7-mm optical zone, the researcher noted higher residual LOA six months after T-PRK and suggested selecting a 6.5 mm or smaller optical zone and using a pupillary diameter in mesopic illumination to select the diameter to avoid patient dissatisfaction [19].

The comparison of pre- and post-HOA values is crucial as T-PRK is now also used to address existing HOAs and LOAs in eyes with refractive errors [6,18]. Thus, the change in individual HOAs six months after T-PRK is a novel finding in our study; this aspect of outcomes is noted rarely in studies [12].

Addressing the existing HOAs is an accepted norm in refractive surgeries to improve the quality of life, especially reducing the issues of glare, halos, and night vision [4]. However, the influence of the pupillary diameter and analysis diameter in addition to the optical ablation zone is crucial since the more central the location of the HOA, the more symptoms will be experienced by patients in less illumination. In our study, we could not find a correlation among these three parameters and HOA-related quality of vision; however, this could be a fertile area for further research.

A number of refractive surgeries are practiced to provide spectacle-free vision. However, to address both LOA and HOAs, wavefront-guided T-PRK has been noted to be more effective and safer than femtosecond LASIK. [19] For both T-PRK and mechanical photorefractive keratectomy, Yildirim et al. noted an increase in the value of HOAs even though the correction of LOAs was successful [20]. It seems that the issue of HOAs after T-PRK, although lower than earlier, is still a matter of concern.

Compared with previous studies, larger optical zones are now used for ablation with modern tools. Seo et al. compared the outcomes of 6-mm and 6.5-mm ablation zones in 2004, while our study compared 7 mm with 6.5 mm and found advantages and disadvantages in relation to addressing HOAs [21].

Single-step T-PRK is recommended to address LOAs effectively in mild to severe myopic eyes [22]. However, both central corneal thickness and pupillary diameter have been found to reduce preoperative HOAs and newly induced HOAs, especially if the anterior surface of the cornea is irregular [23]. In this article, the selection of mild to moderate myopia and excluded cases with thin CCT did not permit us to study the effects of these factors on outcomes in both 6.5-mm and 7-mm optical zone ablation.

In our study, the HOAs were more in eyes that underwent the 6.5-mm optical zone of ablation instead of the 7-mm optical ablation zone, and the pupillary diameter affected this correlation. Night driving and contrast sensitivity are known complaints in patients after refractive surgeries, even if LOA is satisfactorily corrected. HOAs in eyes with a disparity in the optical zone of ablation and pupillary diameter could extenuate these problems in a mesopic setting [9,24]. This result may be due to the fact that we used a suitable pupil diameter and the newer excimer laser with a more advanced ablation profile than that in the past.

In the present study, we compared the impact of selecting smaller optical zones of ablation on changes in HOAs after T-PRK. It would be interesting to study long-term changes through a longer follow-up; there are different refractive surgeries performed by different equipment. A study to review the impact of the tools used for refractive surgery on the HOA would also be of interest.

There were a few limitations in our study. The data are from one institution and managed by one surgeon. Therefore, operator’s variability could pose a challenge while extrapolating the study outcomes to larger populations. The outcomes were based on a six-month follow-up after T-PRK. Corneal healing could take longer. Hence, studies with long-term efficiency and safety are recommended.

## Conclusions

T-PRK is efficient in addressing LOAs. The selection of the optical zone diameter for ablation in T-PRK was not beneficial in addressing HOAs. As such, there is no significant difference in HOAs six months after T-PRK in those managed with the 7-mm and 6.5-mm optical zones, but a decline in HOAs compared to before surgery was noted; especially, the fourth-order spherical aberration in the 6.5-mm group was more than that in the 7-mm group of T-PRK at 6-mm analysis diameter. The pupillary diameter has an independent influence on the changes in the third-order coma type of HOA six months after T-PRK.
